# Factors that influence delaying initial psychiatric treatment in rural Cambodia: A pilot study

**DOI:** 10.1371/journal.pone.0206882

**Published:** 2018-11-01

**Authors:** Akihiro Nishio, Ryo Horita, Toshiyuki Marutani, Mayumi Yamamoto

**Affiliations:** 1 Health Administration Center, Gifu University, Gifu, Japan; 2 Department of Psychopathology, Division of Neuroscience, Graduate School of Medicine, Gifu University, Gifu, Japan; 3 Health Support Center, Tokyo Institute of Technology, Yokohama, Japan; 4 United Graduate School of Drug Discovery and Medical Information Sciences, Gifu University, Gifu, Japan; Rush University Medical Center, UNITED STATES

## Abstract

**Background:**

The WHO reported the gap between the need for treatment and its provision is huge in low- and middle-income countries. It is estimated there are lots of burden to obtain treatment in these countries. This survey intended to show the delay of their first visit to a psychiatric department and the factors that influence the delay. To elucidate the factors affecting medical accessibility for people with mental illness, we propose the concept of duration of untreated mental illness (DUM), which is the duration between the onset or first symptom of mental illness and the first visit to a psychiatric department or clinic.

**Methods:**

Participants were 109 Cambodian adults (18 years old and up) who had a psychiatric consultation in one of the following hospitals. We analyzed the relationships between DUM and patients’ background; age, gender, economic status, education level, occupation, hospital access, and diagnosis.

**Results:**

The average DUM of all participants was 34.8 ± 42.4 months, ranging from 0 to 240 There was no significant difference in DUM by difference in hospital, gender, age, hospital access, education level, occupation, or economic status. Only patient diagnosis was related to DUM. The DUM for patients with schizophrenia and epilepsy was long, while the DUM for patients with neurosis and substance use were short.

**Conclusion:**

To compare DUM with that of other low- and middle-income countries, DUM of our survey is extremely long. However, those reports were from urban areas within the low- and middle-income countries. We considered our report to include a very important sample showing the condition of psychiatric services in rural areas of low-income countries.

## Introduction

The WHO has stated that approximately 76% to 85% of people living in both low- and middle-income countries with severe mental illness receive no treatment for their disorders [1]. Because mental health service systems have not yet adequately responded to the burden of mental disorders, a substantial gap between the need for treatment and available services exists in these countries. Thus, understanding the burden of the people with mental illness who are unable to access psychiatric departments in low- and middle-income countries is an area of needed research. However, it is extremely difficult to identify and recruit these populations. The second-best strategy is to ask psychotic patients who are already connected to psychiatric departments about the factors that influence the delay of their first visit to a psychiatric department. That delay might be influenced by the local access to the hospital, patients’ education, their knowledge of mental illness, and the discrimination of psychotic patients.

To elucidate the factors affecting medical accessibility for people with mental illness, we propose the concept of duration of untreated mental illness (DUM), which is a measurement of the length of time between the onset or first symptom of mental illness and an individual’s first visit to a psychiatric department or clinic. To introduce DUM, we referred to the concept of the duration of untreated psychosis (DUP), which is used as an index for predicting psychotic patients’ prognosis [2] and is used only for psychotic patients. Many reports have suggested that long DUP is a risk factor associated with poor long-term outcomes, such as positive and negative psychotic symptoms, and that shortening DUP might improve long-term recovery [3,4]. Although DUP is usually used for predicting the prognosis of patients with psychosis worldwide, we use DUM here in its original meaning, which is the time between the onset of mental illness and initiation of treatment.

In this study, we compared DUM according to the patients’ backgrounds, including age, gender, education level, occupation, access to the hospital, economic status, and diagnosis. Furthermore, we compared DUP in other countries to the DUP we obtained from the DUM from our survey by excluding the data from individuals that were related to non-psychotic mental illness, including mood disorders, anxiety, and substance use. DUP is a commonly used index in the field of psychiatry, and there are much data from high-income countries and some from low- and middle-income countries. Thus, using DUP would allow for an objective comparison of the burden of visiting the psychiatric department within the targeted area compared to other regions and countries.

The aim of this paper is to collect initial data regarding DUM and to clarify the relationship between DUM and patient’s background in a rural area of Cambodia. To know more about the burden of visiting the psychiatric department, we also investigated the hindrance to continuing treatments for the patients and key individuals with whom the patients first consulted and who most influenced the patients’ decisions to visit a psychiatric service, using a questionnaire.

## Materials and methods

### Targeted country and its health system

We conducted our survey in Siem Reap Province, Cambodia, in March 2014. Cambodia is a country located in the southern part of the Indochina Peninsula in Southeast Asia that has a population of over 15 million. Cambodia has faced many political and economic difficulties. Cambodia is a low-income country, where agriculture remains the dominant economic sector. The gross national income per capita, public-private partnerships (current international $) was 2890 from 2010 to 2014 [5]. Siem Reap Province, the location of our survey, is the 6th largest province in Cambodia, with a population of about 0.9 million in 2008 [6]. Siem Reap had one central hospital in Siem Reap City and three district hospitals in Angkor Chum, Kralanh, and Sout Nikom district. Only the central hospital had two psychiatrists on staff. The other three hospitals provided psychiatric services every week, or every two weeks, with doctors traveling from either the central or another provincial central hospital. There is no available hospitalization setting for psychotic patients in Siem Reap or the neighboring province. Psychiatric services for outpatients in district hospitals have been provided by the NGO of foreign countries. In 2014 throughout the country of Cambodia, only four provincial hospitals provided psychiatric services. Therefore, Siem Reap was exceptional in that psychiatric services were offered at the district hospital. Treatment was free for low-income patients who were recognized through the “identification of poor households program” [7]. At the time of data collection, if patients were judged to be extremely low income, the traffic fee was also paid for patients in the Siem Reap Province.

### Participant’s criteria and data collection

Individuals who were Cambodian people, diagnosed with mental, behavioral and neurodevelopmental disorders by ICD-10, and who had a psychiatric consultation in one of the following hospitals: Siem Reap Referral Hospital, Angkor Chum District Hospital, Kralanh District Hospital, and Soutr Nikom District Hospital in Siem Reap Province were eligible to participate in the study. Participants under 18 years and non- Cambodian people were excluded. DUM and other background data were collected by a Cambodian clinical psychologist using a semi-structured interview.

### Analysis of DUM

We analyzed the relationships between DUM and patients’ background (e.g., age, gender, economic status, education level, occupation, hospital access, and diagnosis). We divided participants into two groups according to age: 20–39 years old and over 40 years old. We also divided the participants into two groups based on socio-economic status: high-income or low-income, according to the “poor identification” carried out by the Cambodian government. We considered monks as belonging to the poor group because they do not generate any income. We divided the participants into three groups based on occupation: unemployed, farmer, and salaried employee. We considered homemakers to be in the “unemployed” category. We divided the participants into six groups based on mental illness: psychosis, mood disorders, neurosis, substance use, epilepsy, and other. Psychosis included schizophrenia and other schizophrenia-like psychotic disorders. Epilepsy was treated within the psychiatric department in Cambodia. Diagnoses were given by a Cambodian psychiatrist, who used either the ICD-10 or DSM-IV as the diagnostic framework. We divided the participants into two groups based on hospital access: those who lived in the same district as the hospital, and those who lived in a different district. Student’s *t*-test was used to compare the DUM according to the patients’ backgrounds.

### Questionnaire

We also asked the patients about the hindrances they faced in continuing their treatment. Our questionnaire had seven items covering financial reasons, traffic problems, family problems, discrimination towards mental illness, ineffective treatment, and other reasons. Multiple choices were presented, and if the participants chose “other reasons,” they were asked to write the reasons in a free-response space.

Furthermore, we asked the patients to name the person with whom they consulted first and the person who most influenced their decision to visit a psychiatric service. Our questionnaire included seven individuals: a family member, monk, village chief, health worker, traditional healer, friend, and other. If the participants chose “other,” they were asked to identify that person in a free-response space.

Finally, we divided the participants into two groups: those for whom the key person was a family member, and those for whom the key person was not. We compared the DUM between each group, using Student’s *t*-test.

### Ethics and data analysis

We obtained written informed consent from the patients who could read. For the patients who could not, Cambodian research assistant obtained verbal consent and checked it in questionnaire after explanation of the survey. No participants received a reward for this study. This research design was approved by the Ethical Review Committee of the Graduate School of Medicine, Gifu University on March 5, 2014 (approval No. 25–369). The statistical analysis was performed using JMP ver. 10.0.2 (SAS Institute, Tokyo, Japan).

## Results

Of 109 participants, 42 were men, and 67 were women. The mean age of the participants was 39.4 ± 13.9 (Men: 36.5 ± 13.3, Women: 41.2 ± 14.0) years old, with a range from 20 to 75 years old. The average DUM of all participants was 34.8 ± 42.4 months, ranging from 0 to 240. Table 1 shows the relationship between DUM and patients’ backgrounds. There were no significant differences in DUM for hospital, gender, age, hospital access, education level, occupation, or economic status. Only patient diagnosis was related to DUM. Significant difference of combination are following; Epilepsy-Other (*p* = .0034), Epilepsy-Mood disorder (*p* = .025), Epilepsy-Neurosis (*p* = .012), Schizophrenia-mood disorder (*p* = .024) and Schizophrenia and Neurosis (*p* = .016). The DUM for patients with schizophrenia and epilepsy was long, while the DUM for patients with neurosis and substance use were short. All the substance users met the criteria for alcohol abuse or dependence. The DUM for the patients with epilepsy was significantly longer than that of the patients with mood disorder, neurosis, substance use, and other disorders. The DUM of the patients with psychosis was significantly longer than that of the patients with mood disorder, neurosis, and substance use.

**Table 1:** Patients’ backgrounds and relationship with DUM.

Background	Sample	Average of DUM	SD	Low 95%	High 95%
	Size				
Place					
Siem Reap Referral Hospital	28	30.5	30.2	18.7	42.2
Angkor Chum district Hospital	24	48.1	62.5	21.7	74.5
Kralanh District Hospital	30	33.9	41.7	18.3	49.5
Soutr Nikom district Hospital	27	28.6	29.7	16.8	40.3
Gender					
Men	42	33.8	36.9	22.3	45.3
Women	67	35.5	45.7	24.3	46.6
Age					
20–39 years old	64	38	47.9	25.8	50.2
More than 39 years old	47	30.7	33.7	20.8	40.6
Access to hospital					
Same district	76	36.3	45.3	26	46.7
Different district	33	31.4	35.1	18.9	43.8
Education					
0 years	44	36.5	36.3	25.4	47.5
1–6 years	40	33.3	50.3	17.2	49.4
More than 6 years	25	34.4	40	17.9	51
Occupation					
Unemployed	25	40.9	37.5	25.4	56.4
Farmer	59	28.1	34.5	19.1	37.1
Salaried employee	25	44.6	59.5	20.1	69.2
Economic status					
Low-income	86	36	43.9	26.4	45.6
High-income	23	31.2	37.6	16	46.4
Diagnosis					
Psychosis	44	47	48	32.5	61.6*
Mood disorder	21	14.2	18	6.1	22.4*
Neurosis	16	18.6	17.4	9.3	27.9*
Substance use	5	10	8.6	-0.7	20.6*
Epilepsy	20	52.7	52.3	28.2	77.2*
Other	3	8.7	13.3	-24.3	41.7*

* Significant difference of combination are following; Epilepsy-Other (p = .0034), Epilepsy-Mood disorder(p = .025), Epilepsy-Neurosis(p = .012), Schizophrenia-mood disorder(p = .024) and Schizophrenia and Neurosis(p = .016).

Table 2 shows the persons with whom the patients consulted first and the individual who most influenced the patients’ decisions seek psychiatric treatment. The results showed that almost all the patients initially consulted their family members. Of the people who most influenced the patients’ decisions to seek psychiatric services, health workers and “others” represented one quarter of the responses. When we asked the participants to identify who the “other” individuals were, almost all of those were nurses or doctors. We analyzed the relationship between DUM and the most influential person. The DUM of the group in which the most influential person was a family member was 35.7 ± 42.2 months. The DUM of the group in which the most influential person was other people (monk, chief of village, health worker, traditional healer, friends and other) was 32.8 ± 43.3 months. There was no significant difference.

**Table 2:** People with whom patients consulted before visiting psychiatric services (N = 109).

	Family Member	Monk	Chief of Village	Health worker	Traditional Healer	Friend	Other
	*n* *(*%)	*n* *(*%)	*n* *(*%)	*n* *(*%)	*n* *(*%)	*n* *(*%)	*n* *(*%)
Person who patients initially consulted	98	1	0	5	1	0	4
	89.9%	0.9%	0.0%	4.6%	0.9%	0.0%	3.7%
Person who most influenced patient’s decision to visit psychiatric service	76	2	3	10	0	4	14
	69.7%	1.8%	2.8%	9.2%	0.0%	3.7%	12.8%

Table 3 shows the barriers to continuing treatment as reported by the patients. Multiple choices were allowed for this question. Our questionnaire had seven items: financial reasons, traffic problems, family problems, discrimination, ineffective treatment, and other. If the participants chose “other,” they were asked to identify the barriers in a free-response space. Forty-four patients reported, “I don’t know any other patient” in typical Cambodian phrasing, so we added this as a reason, as is shown in Table 2. The two remaining “other” reasons were “the doctor will leave,” and “I will go to Thailand.” The results showed the biggest barrier was patient isolation, followed by financial reasons, family problems, and ineffective treatment. Traffic problems and discrimination were not reported to be major obstacles for patients.

**Table 3:** Barriers to continuing treatment for the patients.

Reason	Financial reason	Traffic problem	Too busy	Family problem	Discrimination	Treatment is not effective	No other patient I know	Other
Number	22	3	7	22	2	20	44	2
Percentage (%)	20.2	2.8	6.4	20.2	1.8	18.3	40.4	1.8

## Discussion

There were no significant differences in DUM according to differences in hospital, gender, age, hospital access, education level, occupation, and economic status. Only patient diagnosis was related to DUM. We hypothesized that the knowledge or fear of mental illness differed depending on the disease.

We searched for previous studies analyzing DUP or DUM by patient background in low- and middle-income countries. We found three articles from Pakistan, South Africa, and Hong Kong, and one comparison study between China and Mauritania. Naqvi et al.’s [8] study in Pakistan mentioned no significant association between DUP and gender, marital status, or education. Tomita et al.’s [9] study in South Africa mentioned that young people with lower income, higher education, and a more stable relationship status had longer DUP. There was no significant association between DUP and gender, race/ethnicity, or hospital access. Hui et al.’s [10] study in Hong Kong showed that people with lower age of illness onset and employment status had longer DUP. There was no significant association between DUP and gender, education, marital status, hospital access, living status, smoking, or family history of mental illness. On the other hand, Thankoor’s [11] report is very unique, in that it compared DUM (they include mood disordered patients as participants) between Changsa, China, and Beau Bassin, Mauritius. Although the authors did not directly show the length of DUM, they divided their participants into 2 groups: short and long DUP, and they analyzed the effects of gender, marital status, diagnosis, education, economic status, insurance coverage, and awareness of stigma towards mental illness. They reported a correlation between long DUM and low monthly income in Mauritania.

Similar to the report from Pakistan, we could not find any difference in DUM by economic status, but this was in contrast to the reports from South Africa and Mauritania. Since the poor identification insurance system started in 2010 in Siem Reap Province, conditions have changed for patients visiting psychiatric services for the first time. We neglected to measure those changes in our survey. Additionally, the poor identification insurance system was bankrupted in Siem Reap Province in 2017; therefore, economic status might influence DUM for new patients in the next step of our survey.

Similar to the South African report, no significant impact of education on DUP or DUM was found in our survey. The South African report indicated high education lengthened DUP. Both reports showed hospital access did not influence DUP. In any case, we should accumulate such data from various countries to understand patients’ decision-making process in seeking psychiatric services.

To compare DUP with that of other countries, we calculated mean DUP from DUM. The mean DUP was the mean DUM of the patients with psychosis in our study. The mean DUP of our study was 47.0 ± 48.0 months; in other words, 188.0 ± 192.0 weeks. Bora et al. [12] carried out a meta-analysis of DUP and selected 27 surveys from high-income countries and Shanghai, China. According to their report, the mean DUP was from 9.3 to 147.2 weeks. In their survey, the quantiles of 25% and 75% were 36.9 and 77.9 weeks, respectively. Therefore, we can estimate the standard DUP in high-income countries was around 50 weeks. The DUP in our survey was about 4 times longer than that of high-income countries. The reports showing mean DUP from low- and middle-income countries are few. As we saw, there are only eight articles which showed the DUP for each country [8, 10, 13–18]. Table 4 shows the characteristics of the surveys and DUP.

**Table 4:** Characteristics of existing research and samples examining mean DUP.

Ref.	Author	Publication Year	Location	Diagnosis of Interest	Mean DUP
13	Apiquian	2002	Mexico City, Mexico	Psychosis	59.5 weeks
14	Oosthuizen	2005	Cape Town, South Africa	Psychosis	32.7 ± 51.3 weeks
15	Ayres	2007	São Paulo, Brazil	Psychosis	51 weeks
8	Naqvi	2009	Karachi, Pakistan	Schizophrenia	56.0 ± 117.6 weeks
16	Sharifi	2009	Teheran, Iran	Psychosis	52.0 ± 114.7 weeks
17	Burns	2010	KwaZulu-Natal, South Africa	Psychosis	35.1 ± 62.0 weeks
18	Fayes	2014	Riyadh, Saudi Arabia	Psychosis	22.7 ± 16.9 weeks
10	Hui	2015	Hong Kong, China	Psychosis	73.6 weeks
Our survey	Nishio		Siem Reap, Cambodia	Psychosis	188.0 ± 192.0 weeks

Compared to the above data from developing countries, the DUP of our survey was extremely long. However, those reports were from urban areas within the low- and middle-income countries. On the other hand, our survey was carried out in rural areas of low-income country. We considered our report to include a very important sample showing the condition of psychiatric services in rural areas of low-income countries.

We also asked questions regarding who the patient initially reached out to about their condition and who were the most influential in the patients’ decisions to visit psychiatric services. The most influential people in the decision-making process were the patients’ family members, in almost all cases, and they were often the first people contacted by the patients to seek help. Padilla et al. [19] reported that the intervention that provided knowledge about mental illness dramatically decreased DUP in Argentina. Therefore, it is considered effective to provide knowledge about mental health to family members, that is to say the general population, in order to shorten DUP or DUM.

We considered that DUP or DUM could be an index to evaluate the mental health system in rural areas. The knowledge about mental health, access to the hospital, insurance system and clinical level in the area might influence the DUM or DUP. We should accumulate more data, considering such back ground of health system.

### Limitations

There were several limitations to this study. First, the targeted sample was not large. Second, we obtained our data from patients who had already selected a psychiatric service. Therefore, patients who had visited the hospital for a long time were included along with those who had only visited for a short time. Thus, our DUM may not show the actual status in Siem Reap, since the situations of local hospital access, patient education, knowledge of mental illness, and the discrimination of psychotic are time dependent. We recommend that the participants of future research should be limited to only new patients.

However, we considered our report to be extremely important. Our study was the first report of DUM/DUP and showed the relationship between DUM and patients’ backgrounds in rural areas of low-income countries. We are convinced this report provides a great deal of knowledge that will enhance the further development of well-structured surveys and mental health services in low- and middle-income countries.
